# One-carbon metabolic pathway is a novel molecular signature for *CD44*-positive intestinal-type gastric cancer

**DOI:** 10.1038/s41420-025-02704-5

**Published:** 2025-08-23

**Authors:** Seyeon Joo, Yoojin Bae, Bo Kyung Yoon, Yeonjin Je, Yuna Kim, Minki Kim, Su-Jin Shin, Sungsoon Fang, Jie-Hyun Kim

**Affiliations:** 1 Graduate School of Medical Science, Brain Korea 21 Project, Yonsei University College of Medicine, Seoul, South Korea; 2 Department of Biomedical Sciences, Gangnam Severance Hospital, Yonsei University College of Medicine, Seoul, South Korea; 3 Department of Internal Medicine, Gangnam Severance Hospital, Yonsei University College of Medicine, Seoul, South Korea; 4 Department of Microbiology and Immunology, Yonsei University College of Medicine, Seoul, South Korea; 5 Institute for Immunology and Immunological Diseases, Yonsei University College of Medicine, Seoul, South Korea; 6 Department of Medicine, Yonsei University College of Medicine, Seoul, South Korea; 7 Department of Pathology, Gangnam Severance Hospital, Yonsei University College of Medicine, Seoul, South Korea

**Keywords:** Gastric cancer, Tumour heterogeneity, Transcriptomics, RNA sequencing

## Abstract

Intratumoral heterogeneity (ITH), arising from various factors, plays a crucial role in diverse cancers, yet research on ITH remains in its early stages. To explore this phenomenon further, we conducted single-nuclei transcriptome profiling on patient-derived organoids (PDOs) from two histologically pure subtypes of gastric cancer. We identified differences in cancer stem cell marker expression among intestinal-type samples and their correlation with one-carbon (1C) metabolism. Notably, some gastric cancer samples, although histologically classified as intestinal-type, exhibited diffuse-like genetic characteristics. This finding suggests the potential for employing a 1C metabolism inhibition strategy as a therapeutic approach for these genetically diffuse-like gastric cancers. This study highlights the necessity of addressing ITH in PDO-based preclinical models and contributes valuable insights toward advancing precision medicine treatments for gastric cancer.

## Introduction

Gastric cancer (GC) ranks as the fifth most prevalent cancer globally and the fourth leading cause of cancer-related mortality among solid tumors, though its prognosis remains poor [1–3]. For diagnosis, several classification criteria are used, with Lauren’s criteria serving as the standard for histologic classification, categorizing GC into intestinal- and diffuse-types [4–7]. The diffuse-type, typically found in women and younger patients, is characterized by single cells or loosely connected cells infiltrating the gastric wall [2, 8, 9]. In contrast, the intestinal-type, which is more commonly found in older patients and men, shows glandular, solid, or intestinal structures, including tubular formations, and is frequently associated with environmental factors like *Helicobacter pylori* infection [10–12]. Recent research indicates that while the intestinal-type is considered genomically more unstable, the diffuse-type is more invasive and displays more aggressive traits in cancer progression [13, 14].

Intratumoral heterogeneity (ITH) describes the presence of genetically diverse subpopulations of tumor cells within a single tumor, either intermingled or spatially separated. This diversity results in variations in tumor cell growth, immune response, metabolism, and metastatic potential [15–17]. ITH is a well-documented phenomenon in various cancers, including GC [18, 19]. Although significant research has confirmed its existence, more in-depth studies are still required to explore its impact on treatment strategies [20].

One-carbon (1C) metabolism, comprised of folate metabolism and methionine metabolism, is known to serve as an important mechanism in regulating cancer cells [21]. 1C metabolism is generally upregulated in cancer, primarily because cells require one-carbon units for nucleotide synthesis, methylation reactions, and for the generation of reducing cofactors [22, 23]. Cancer cells utilize the outputs obtained from these folate and methionine cycles to supply energy to cancer cells, facilitate cancer cell proliferation through nucleic acid synthesis, regulate cancer cell fate through redox control, and maintain homeostasis [24–26]. A significant number of 1C metabolism genes play crucial roles in cancer proliferation and survival through purine production. Additionally, drugs such as Methotrexate and 5-fluorouracil (5-FU), which have been used for cancer patients for decades and remain actively utilized today, function by inhibiting key genes within 1C metabolism. The clinical efficacy of these therapies against various cancers clearly demonstrates that cancer cells are highly dependent on 1C metabolism [27, 28].

Although various metabolic pathways contribute to cancer pathogenesis, 1C metabolism has attracted attention as a potential therapeutic target due to its observable upregulation in cancer patients. Recent studies have demonstrated a close association between GC and 1C metabolism, and while numerous investigations are currently underway regarding 1C metabolism in GC, further in-depth research remains necessary [29, 30].

Patient-derived organoids (PDOs) are three-dimensional models created from tumor cells taken from patients’ primary tumors [31]. They effectively replicate the tissue architecture and cellular composition of the original tumor [32–34]. PDOs allow researchers to perform individualized tumor response testing, which plays a key role in discovering potential predictive biomarkers [35–38]. This study utilized single-nucleus transcriptomics to examine ITH in PDOs from two GC subtypes: intestinal- and diffuse-types [39]. While sequencing analyses of PDOs may not completely capture the spectrum of tumor cell subpopulations found in the primary tumor, we addressed this limitation by validating our results with single-cell RNA sequencing data from human tissue.

Our objective is to leverage the findings from this study to confirm consistent features of ITH in both PDOs and human samples. By examining both histological classifications and genetic expression differences in histologically pure GC types, we aim to develop effective treatment strategies.

## Results

### Genetic features of organoids derived from clonal samples of gastric cancer tissue

**Fig. 1:**
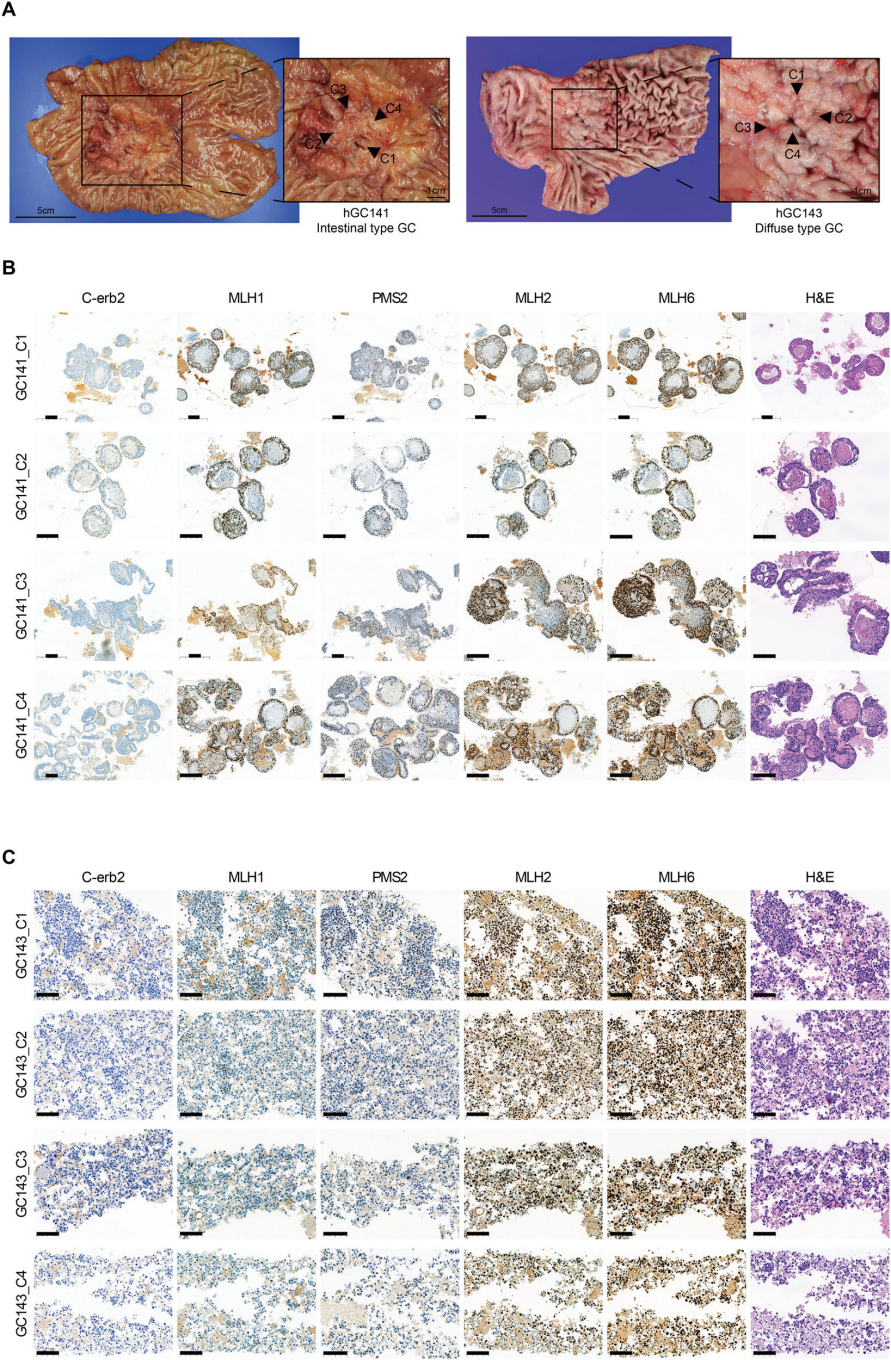
Clonal selection sites and genetic profiling of organoids derived from gastric cancer tissue. **A** Intestinal- and diffuse-type gastric cancer specimens marked with regions selected for clonal sampling. **B**, **C** Immunohistochemical experiments performed on organoids derived from each clonal sample of gastric cancer specimens, GC141 and GC143, including H&E staining. Scale bars: **B** 200 μm, **C** 100 μm.

Tissue samples were obtained from patients diagnosed with pure intestinal- and pure diffuse-type GC, and four distinct regions were collected from each sample for organoid culture. Organoids were successfully cultured from all eight samples (Fig. 1A). To assess their genetic characteristics, we performed staining on the cultured organoids. The staining results showed that all eight samples were positive for *MLH1*, *PMS2*, *MLH2*, and *MLH6*, classifying them as microsatellite stable. Additionally, *C-erb2* was found to be negative in all eight samples (Fig. 1B, C).

### Diffuse- and intestinal-type gastric cancers display unique molecular characteristics

**Fig. 2:**
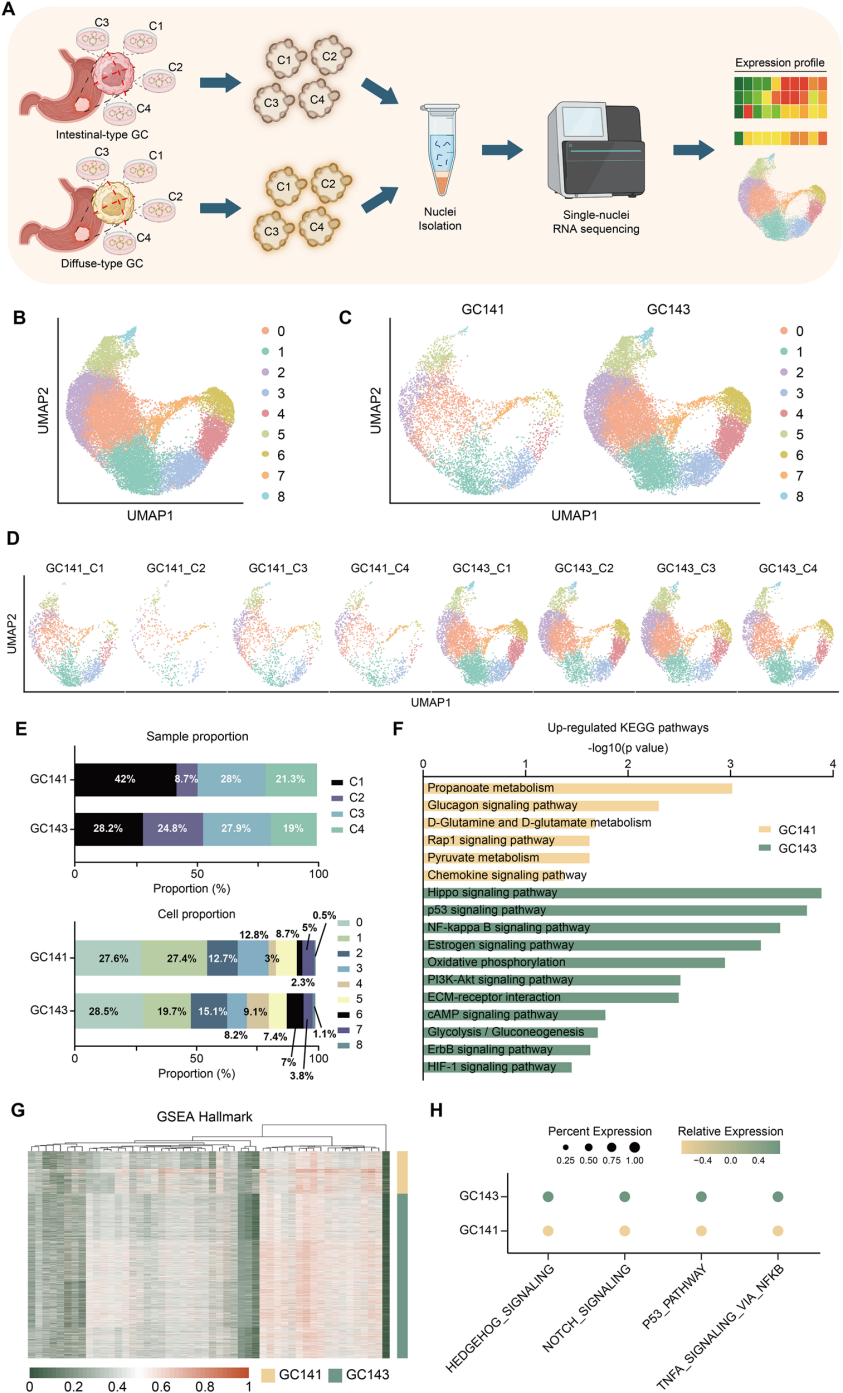
Distinct molecular characteristics of intestinal and diffuse subtypes in gastric cancer. **A** Schematic illustration of patient-derived organoid generation and snRNA-seq analysis. **B** Uniform Manifold Approximation and Projection (UMAP) plots displayed nine individual clusters from 24,340 single cells. **C**, **D** The distribution of annotated cell clusters shown on the UMAP, divided into two subtypes, with each cluster distinguished by clonal samples from the respective subtypes. **E** The cell proportion of clonal samples and nine clusters of each subtype. **F** Comparison of KEGG pathway enrichment results for DEGs in each subtype. **G** Comparison of Hallmark pathway enrichment between the two subtypes using Gene Set Enrichment Analysis (GSEA). **H** Dotplot of cancer stem cell-related pathway expression between the two subtypes.

After collecting tissue samples from two subtypes of GC, we extracted four different regions from each subtype. We were able to successfully culture organoids from all eight samples, and nuclei were isolated from the resulting organoid samples. Single-nucleus RNA sequencing was performed on the nuclei isolated from PDOs derived from four regions of intestinal-type (GC141_C1-4) and four regions of diffuse-type (GC143_C1-4) GC tissues (Fig. 2A). Nine transcriptomic clusters were identified through analysis and projected into two-dimensional space using UMAP. Visualization by histological subtype and clonal origin revealed that cells were uniformly distributed across all conditions (Fig. 2B–D). When examining cell proportions across clusters within the two subtypes, we found no significant differences between the two samples. Similarly, the proportions of clonal samples within each subtype showed minimal variation, although the number of cells in cluster C2 was notably lower in GC141 (Fig. 2E). Through pathway analysis, we observed differences in enriched pathways between the pure intestinal- and pure diffuse-type, as identified using both KEGG and Gene Set Enrichment Analysis (GSEA) HALLMARK gene sets (Fig. 2F, G). Diffuse-type GC is well-known for its high malignancy and elevated stemness. Our data confirmed this, revealing a significant upregulation of cancer stem cell-related pathways in the diffuse-type GC sample GC143, aligning with established findings (Fig. 2H). These results highlight that GC141 and GC143 not only differ histologically as pure intestinal- and pure diffuse-type but also exhibit distinct molecular characteristics.

### Intratumoral heterogeneity in intestinal-type gastric cancer correlates with high *CD44* expression and 1C metabolism enrichment

**Fig. 3:**
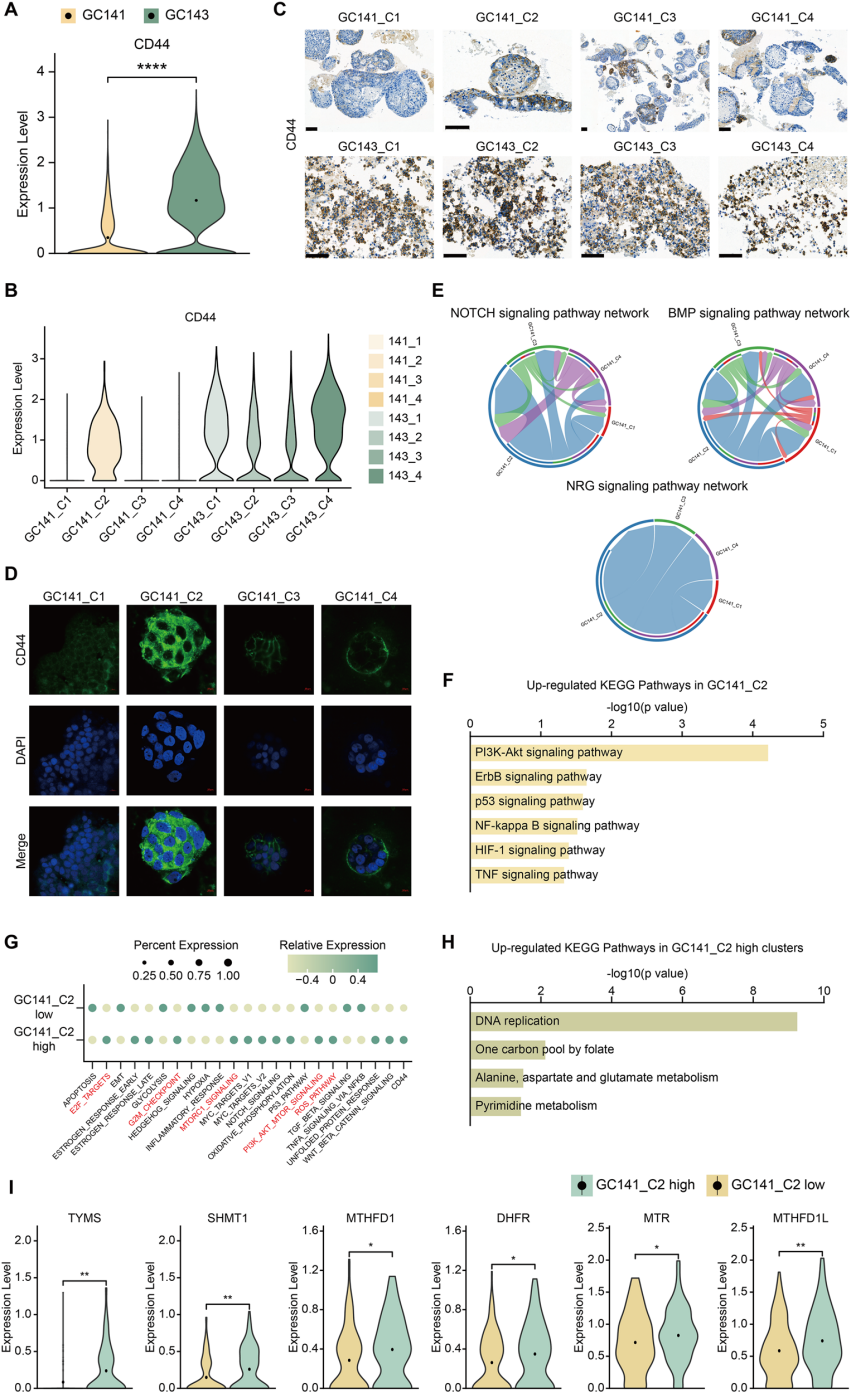
Intratumoral heterogeneity of intestinal-type gastric cancer and 1C metabolism enrichment in *CD44*-high clusters of intestinal-type gastric cancer. **A** Vlnplot of *CD44* expression between the two subtypes and **B** each clonal samples. **C** Immunohistochemical experiments for *CD44* staining were performed on each clonal samples from the two subtypes. Scale bars: 100 μm. **D** Immunocytochemistry of GC141 clonal sample organoids to show the expression of *CD44* (Alexa488, green). DAPI (blue) was used for nuclear staining. **E** ‘CellChat’ chord diagram showing ligand-receptor pairs and their weights contributing to signalings among clonal samples of intestinal-type GC, GC141. **F** KEGG pathway enrichment analysis of identified DEGs in GC141_C2 compared to other clonal samples of GC141. **G** Dotplot showing pathway enrichment of ‘*CD44*-high’ and ‘*CD44*-low’ expressing clusters of GC141_C2. **H** KEGG pathway enrichment analysis of identified DEGs in ‘*CD44*-high’ expressing clusters compared to ‘*CD44*-low’ expressing clusters of GC141_C2. **I** Vlnplot displaying the expression of 1C metabolism-related genes between ‘*CD44*-high’ and ‘*CD44*-low’ clusters of GC141_C2. Statistical comparisons were performed using two-tailed Student’s *t*-test (*P < 0.05, **P < 0.01, ***P < 0.001, ****P < 0.0001, ns not significant).

Diffuse-type GC, a major histological subtype, displays a highly invasive phenotype with pronounced malignant and stemness-associated characteristics [40, 41]. Additionally, as previously mentioned cancer stem cell-related pathways were significantly enriched in the diffuse-type GC sample GC143. To investigate this further, we focused on the expression of *CD44*, a well-established marker of cancer stem cells. *CD44* is widely recognized as a key marker for cancer stem cells in various solid malignancies, including GC, and has been extensively studied as both a tumor biomarker and a therapeutic target [42]. As anticipated, *CD44* expression was significantly higher in GC143 compared to GC141, a trend that was consistent across all clonal samples (Fig. 3A). Similarly, in all clonal samples of GC143, *CD44* expression was elevated. Interestingly, one clonal sample from GC141, specifically GC141_C2, exhibited *CD44* levels comparable to those in GC143 (Fig. 3B). When comparing *CD44* expression between clusters of GC141 and GC143, *CD44* was elevated in most clusters of GC143. However, some clusters of GC141 also exhibited clear expression of *CD44* (Supplementary Fig. 1A). Given the well-known ITH of GC, we performed IHC staining for *CD44* on all eight organoid clonal samples to validate these findings from single-nuclei transcriptomic analysis [43]. The staining revealed strong signals in all clonal samples of GC143, with GC141_C2 also showing a relatively strong signal, further confirming the presence of ITH (Fig. 3C). Interestingly, consistent results were also observed in *CD44* staining of GC tissues. While *CD44* expression was generally high across all areas in GC143, GC141 exhibited regions with high *CD44* expression alongside areas with lower expression, reflecting the ITH characteristic of GC141 (Supplementary Fig. 1B). Additionally, immunocytochemistry in GC141 organoids confirmed the high *CD44* expression in GC141_C2 (Fig. 3D). Since both transcriptomic analysis and experimental validation indicated elevated expression of stemness-related genes in GC141_C2, we used ‘CellChat’ to further analyze the increased communication signals between this *CD44*-high clonal sample and other clonal samples of intestinal-type GC, GC141 [44]. Among the four clonal samples, GC141_C2 showed significant enrichment in cancer stem cell-related pathway networks, including the ‘NOTCH signaling pathway,’ ‘BMP signaling pathway,’ and ‘NRG signaling pathway,’ compared to the other clonal samples (Fig. 3E). To further explore the enriched pathways in GC141_C2, we conducted a deeper analysis by subsetting GC141 and comparing the differentially expressed genes (DEGs) of GC141_C2 with those of the other clonal samples. Cancer stem cell-related pathways were significantly enriched in KEGG analysis and GC141_C2 also displayed enriched pathways similar to those upregulated in GC143, indicating its diffuse-like characteristics (Fig. 3F, Supplementary Fig. 1C, D). To gain deeper insights into the stemness-related molecular characteristics of GC141_C2, we isolated clusters corresponding to GC141_C2 and divided them into high and low groups based on *CD44* expression. The highly *CD44*-expressing clusters in GC141_C2 exhibited increased activity in 1C metabolism-related pathways (Fig. 3G). Subsequent DEG analysis between ‘GC141_C2 high’ and ‘GC141_C2 low’ revealed that, among all pathways exceeding the significance threshold for p-values, key 1C metabolism-related pathways—such as ‘DNA replication,’ ‘One carbon pool by folate,’ ‘Alanine, aspartate, and glutamate metabolism,’ and ‘Pyrimidine metabolism’—were prominently enriched (Fig. 3H, Supplementary Fig. 1E). DNA replication, essential for cell division and transmission of genetic information, is closely linked to the folate and methionine cycles—core components of 1C metabolism [27, 45]. Both our KEGG and HALLMARK analyses consistently revealed enrichment of proliferation and cell cycle pathways, which are critically dependent on 1C metabolism. This finding further supports that the ‘GC141_C2 high’ population is characterized by enhanced 1C metabolic activity (Supplementary Fig. 1F). Additionally, a comparison of 1C metabolism-related gene expression showed that most genes, including *TYMS* and *SHMT1*, were highly expressed in ‘GC141_C2 high’ (Fig. 3I). These findings underscore the clear presence of ITH within intestinal-type GC and highlight the enrichment of 1C metabolism pathways in samples with elevated cancer stem cell-related pathways.

### Diffuse-like intestinal-type gastric cancer exhibits a positive correlation between *CD44* expression and 1C metabolism

Trajectory analysis is a powerful approach that reconstructs the dynamic progression of cellular states along a pseudotime axis, enabling the inference of differentiation hierarchies, cell state transitions, and underlying gene regulatory mechanisms. While trajectory analysis has been widely used in stem cell biology to delineate lineage hierarchies, its application has expanded to broader contexts, including the identification of key fate-determining regulators and the prediction of disease progression.

**Fig. 4:**
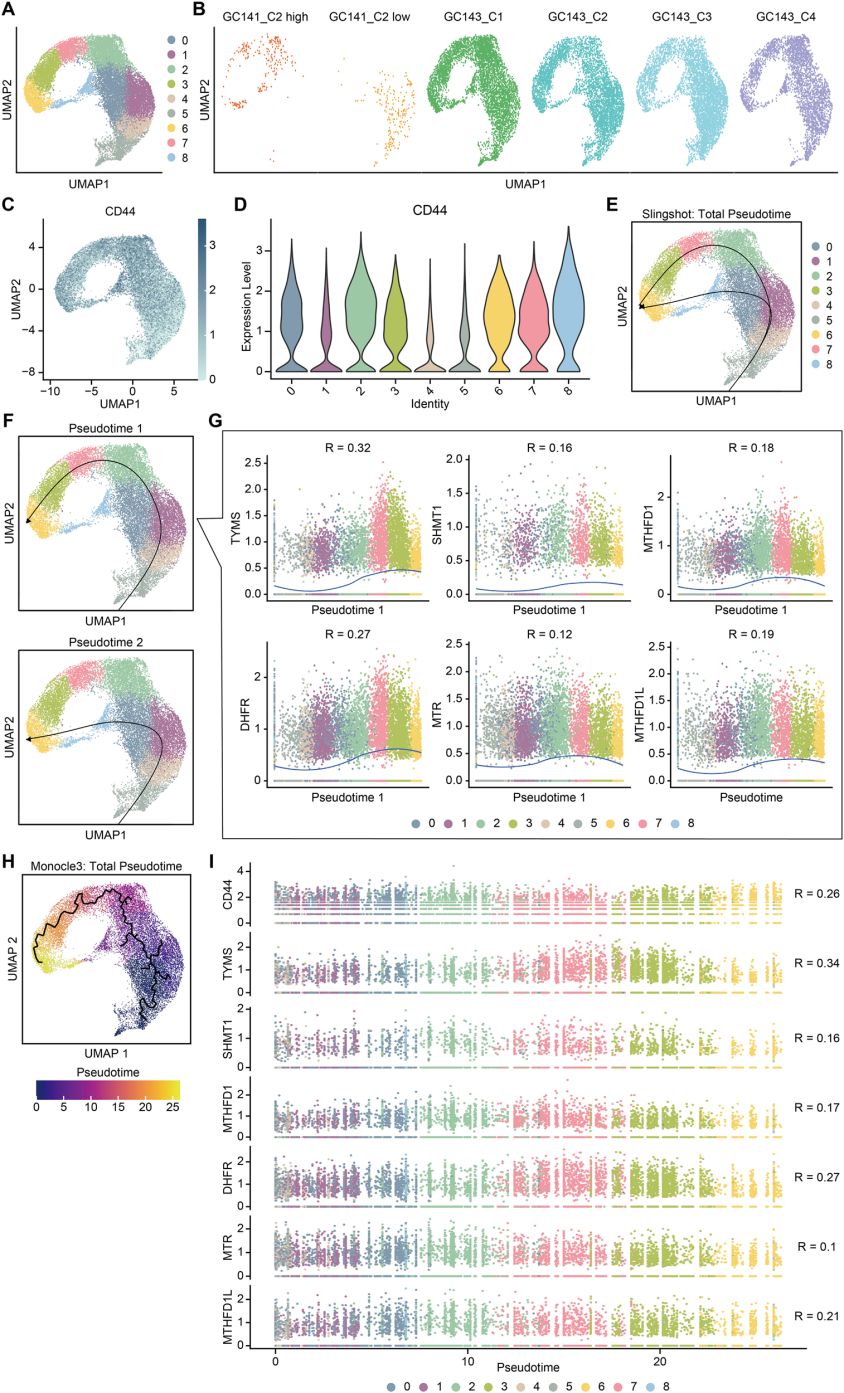
Trajectory analysis identifies positive correlation between *CD44* expression and 1C metabolism in diffuse-like intestinal-type gastric cancer. **A** UMAP plot showing nine individual clusters. **B** UMAP plots displaying ‘*CD44*-high’ and ‘*CD44*-low’ clusters of GC141, alongside the clonal samples of GC143. **C** Dimplot visualizing *CD44* expression. **D** Vlnplot showing expression of *CD44* within each clusters. **E**, **F** Prediction of trajectories using ‘Slingshot’ analysis; ‘Slingshot’ results illustrating the two individual trajectories of lineages as lines, with arrows indicating the direction of pseudotime progression. **G** Scatterplots depicting the positive correlation levels of 1C metabolism-related genes along the pseudotime 1 axis. Dots: single cells; colors: clusters. (*R > 0.1). **H** UMAP plot of trajectory analysis performed using ‘Monocle3.’ **I** Scatterplots representing the positive correlation of 1C metabolism-related gene expression with pseudotime in ‘Monocle3’ trajectory analysis.

Given that GC141_C2 exhibits diffuse-like genetic characteristics despite being histologically classified as pure intestinal-type, we performed a trajectory analysis using ‘Slingshot’ [46]. For accuracy, this analysis included four clonal samples from GC143 and two clusters from GC141_C2, categorized into high and low groups based on *CD44* expression, resulting in a total of six samples (Fig. 4A, B). We utilized ‘Slingshot’ to infer the developmental trajectories within these combined clusters, focusing on the relationship between diffuse-like GC141_C2 subpopulations and pure diffuse-type GC143_C1-4.

Considering the strong relevance of trajectory analysis to stem cell biology, we reasoned that tracing a lineage from a cluster with low expression of the stemness marker *CD44* to clusters with higher *CD44* expression would be biologically meaningful. To ensure accurate reconstruction of the stemness-related differentiation trajectory, we designated Cluster 5, which exhibited the lowest *CD44* expression, as the starting point of the analysis (Fig. 4C, D). Starting with this cluster, the analysis revealed two distinct lineages along pseudotime progression (Fig. 4E). Both lineages, pseudotime 1 and pseudotime 2, showed a trajectory from clusters with low *CD44* expression to those with higher *CD44* expression (Fig. 4F). Further examination of key 1C metabolism genes along pseudotime 1 revealed that most genes, including *TYMS*, *DHFR*, and *SHMT1*, exhibited a positive correlation with the increase of *CD44* expression (Fig. 4G).

To further validate our findings, we conducted an additional trajectory analysis using ‘Monocle3,’ an alternative pseudotime inference tool [47]. To ensure consistency with the ‘Slingshot’ analysis, we again designated Cluster 5, with the lowest *CD44* expression, as the root. ‘Monocle3’ revealed a similar lineage trajectory to that identified in ‘Slingshot’ pseudotime 1, reinforcing the robustness of our initial results (Fig. 4H). Notably, cells along this lineage also exhibited a positive correlation with the expression of genes involved in the 1C metabolic pathway (Fig. 4I). These consistent results across independent trajectory inference methods support the notion that increased *CD44* expression is associated with upregulation of 1C metabolism genes, suggesting a potential link between stemness and metabolic reprogramming.

### *CD44*-high expressing intestinal-type gastric cancer displays enhanced 1C metabolism pathways compared to *CD44*-low expressing intestinal-type gastric cancer

**Fig. 5:**
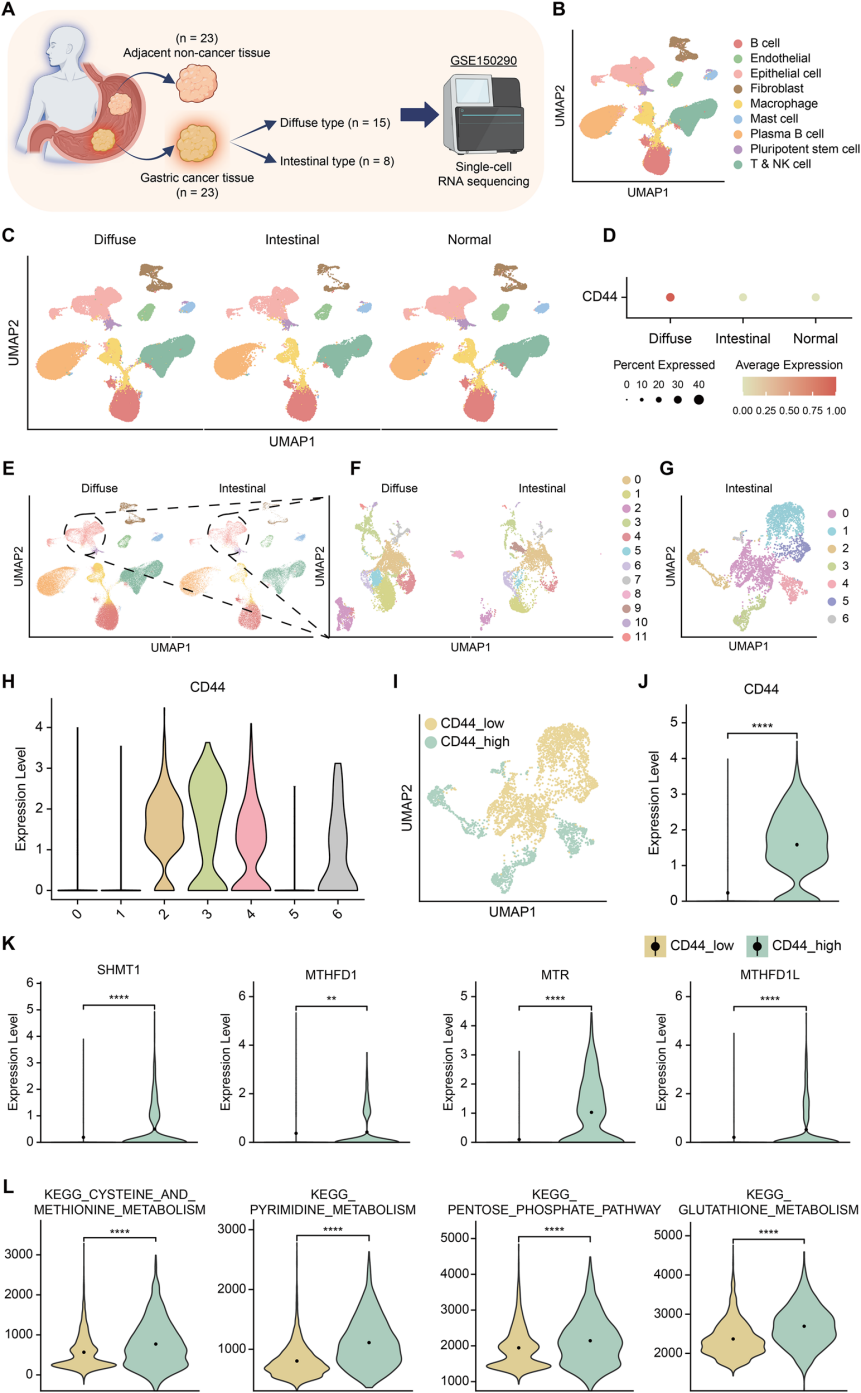
Validation of intratumoral heterogeneity and *CD44*-related 1C metabolism pathways in human intestinal-type gastric cancer via single-cell transcriptomics. **A** Schematic illustration of single-cell RNA sequencing public data consisting of two subtypes of human gastric cancer tissue, diffuse- and intestinal-type. **B**, **C** UMAP plots displayed nine individual clusters from 195,263 single cells with three types of samples, diffuse, intestinal, and normal. Annotation was done through ‘SingleR’ package. **D** Dotplot showing *CD44* expression levels across diffuse- and intestinal-type gastric cancers, as well as normal samples. **E** UMAP of diffuse- and intestinal-type clusters subsetted from (**C**), with a total of 107,389 cells. **F** UMAP of epithelial cell type clusters, derived from (**E**), encompassing a total of 12,351 cells with 12 clusters. **G** UMAP of epithelial cell clusters from the intestinal-type clusters, extracted from (**F**), comprising a total of 3898 cells with seven clusters. **H** Vlnplot showing *CD44* expression levels across seven clusters of intestinal epithelial cell types. **I** UMAP of clusters categorized into ‘*CD44*_high’ and ‘*CD44*_low’ expressing groups as shown in (**H**). **J** Vlnplot depicting *CD44* expression levels of clusters categorized into ‘*CD44*_high’ and ‘*CD44*_low’ groups. **K** Vlnplot illustrating the expression levels of 1C metabolism-related genes in ‘*CD44*_high’ and ‘*CD44*_low’ expressing groups. **L** Vlnplot demonstrating the enrichment levels of 1C metabolism-related KEGG pathways in the ‘*CD44*_high’ and ‘*CD44*_low’ expressing groups. GSEA KEGG pathway analysis was performed using the ‘escape’ package. Statistical comparisons were performed using two-tailed Student’s *t*-test (*P < 0.05, **P < 0.01, ***P < 0.001, ****P < 0.0001, ns not significant).

To validate the findings from the transcriptomic analysis of organoids derived from human GC tissue, we analyzed publicly available single-cell transcriptome sequencing data from 23 GC patients, each with both adjacent normal and GC tissues [48]. After performing quality control and annotation, we generated a UMAP that categorized the cells into nine distinct types (Fig. 5A, B, Supplementary Fig. 2A). We observed that all cell types were uniformly distributed across diffuse-type, intestinal-type, and normal tissues (Fig. 5C). Notably, *CD44*, a cancer stem cell marker, showed the highest expression in diffuse-type samples (Fig. 5D). To further compare diffuse and intestinal subtypes, we isolated the samples corresponding to each subtype and focused our analysis on epithelial cells by subsetting the epithelial cell cluster (Fig. 5E, F). When examining *CD44* expression, it was found to be higher in diffuse-type GC, but elevated *CD44* expression was also observed in certain areas of intestinal-type GC (Supplementary Fig. 2B). To explore the characteristics of ITH and the correlation between *CD44* and 1C metabolism in intestinal-type cells, we further segregated the intestinal-type cells for analysis (Fig. 5G). Examination of *CD44* expression in intestinal-type samples revealed that only four out of seven clusters exhibited high *CD44* expression. We then restructured these clusters into ‘*CD44*_high’ and ‘*CD44*_low’ groups, following the same approach as in previous analyses (Fig. 5H, I). Despite the relatively smaller number of cells in the ‘*CD44*_high’ cluster, *CD44* expression, along with other cancer stem cell marker genes such as *LGR5*, *ALDH1A1*, and *FUT4*, was significantly higher compared to the ‘*CD44*_low’ cluster (Fig. 5J, Supplementary Fig. 2C). Similarly, key 1C metabolism genes, including *SHMT1*, *MTHFD1*, *MTR*, *MTHFD1L, PHGDH, PSAT1, and PSPH*, also showed significantly higher expression in the ‘*CD44*_high’ cluster (Fig. 5K, Supplementary Fig. 2D). GSEA analysis between the two clusters further confirmed that multiple 1C metabolism-related KEGG pathways, such as ‘KEGG_CYSTEINE_AND_METHIONINE_METABOLISM,’ ‘KEGG_PYRIMIDINE_METABOLISM,’ ‘KEGG_PENTOSE_PHOSPHATE_PATHWAY,’ ‘KEGG_GLUTATHIONE_METABOLISM,’ ‘KEGG_DNA_REPLICATION,’ and ‘KEGG_FOLATE_BIOSYNTHESIS,’ were significantly enriched in the ‘*CD44*_high’ cluster, consistent with the PDO transcriptome analysis (Fig. 5L, Supplementary Fig. 2E). These results confirm that, consistent with the PDO transcriptome analysis, the human intestinal-type GC transcriptome analysis also reveals ITH and a positive correlation between *CD44* expression and 1C metabolism-related genes and pathways.

### 1C metabolism inhibitors may be an effective treatment for *CD44*-high intestinal-type gastric cancer

**Fig. 6:**
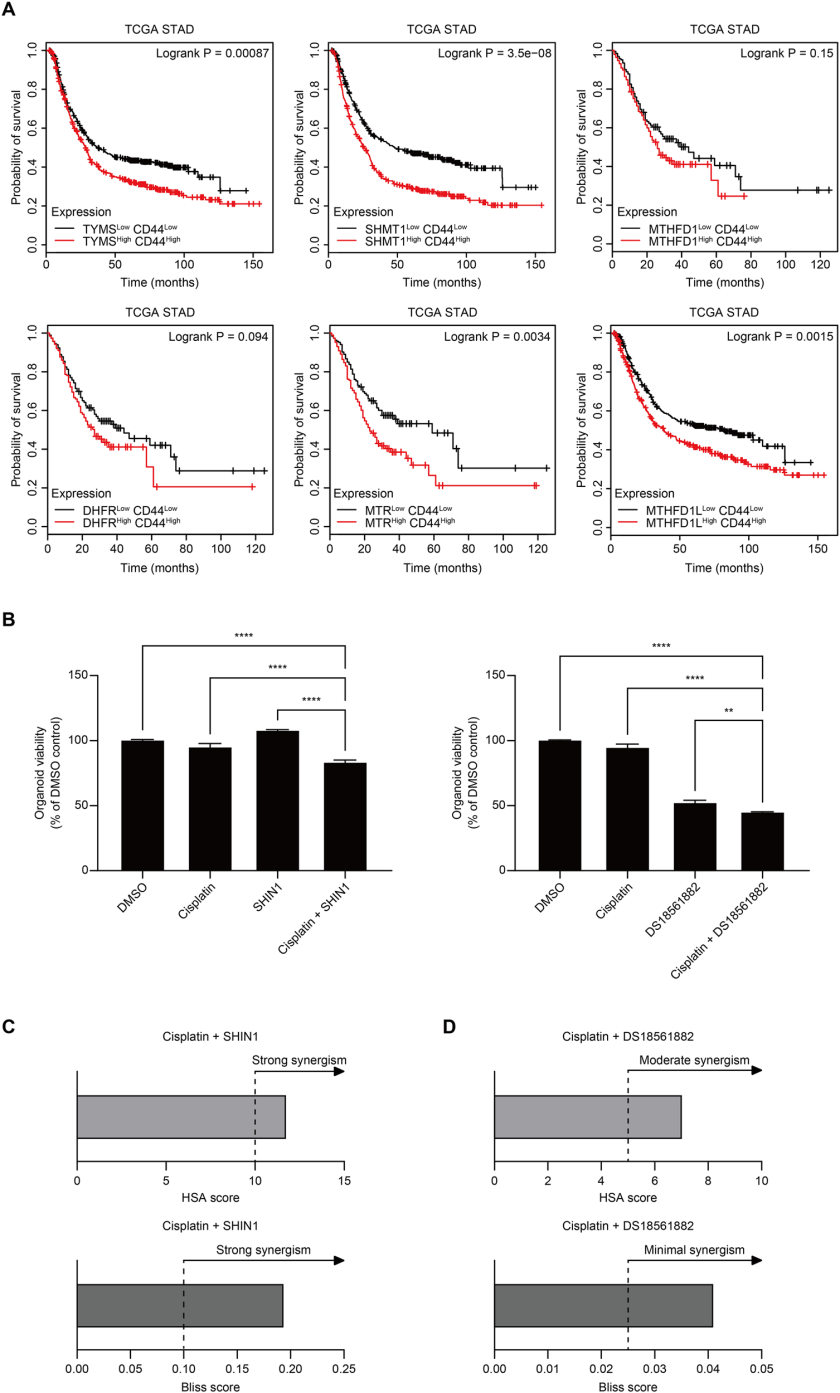
Validation of 1C metabolism inhibitors in patient-derived organoid and TCGA data reveals potential for enhanced therapeutic strategies in gastric cancer. **A** Kaplan–Meier survival curves for 1C gene^high^/*CD44*^high^ and 1C gene^low^/*CD44*^low^ groups in the TCGA stomach adenocarcinoma cohort. The p-values were calculated by the Log-rank test. **B** Organoid viability of GC141_C2 organoid in response to 1C metabolism inhibitors and chemotherapeutic drugs (cisplatin 3 μM; SHIN1 15 μM; DS18561882 40 μM). **C**, **D** The synergistic effects of multiple drugs were validated using the HSA and Bliss models (HSA scores ≥10 indicate strong synergism, ≥5 moderate synergism; Bliss scores, typically ranging between −1 and 1, are interpreted as strong synergism if ≥0.1 and minimal synergism if ≥0.025). Statistical comparisons were performed using ANOVA (*P < 0.05, **P < 0.01, ***P < 0.001, ****P < 0.0001, ns not significant).

To explore the relationship between *CD44* and 1C metabolism in patient samples, we analyzed TCGA-STAD data using ‘GEPIA’ and ‘Kaplan–Meier plotter’ [49–52]. Our results demonstrated that high co-expression of *CD44* and 1C metabolism-related genes was associated with poorer survival outcomes (Fig. 6A). This trend underscores the clinical relevance of the positive correlation between *CD44* and 1C metabolism-related genes.

To further confirm these findings at the PDO level, we treated the GC141_C2 organoid with 1C metabolism inhibitors and compared their effects with those of commonly used chemotherapy drugs. Additionally, to assess the combinatorial efficacy of the two drugs, we treated cells with both agents simultaneously and evaluated whether the combined treatment enhanced the inhibitory effect compared to each agent alone. We used ‘SHIN1,’ which inhibits *SHMT1/2*, and ‘DS18561882,’ which targets *MTHFD1/2*, as the inhibitors of 1C metabolism. For the chemotherapy agents, we selected cisplatin and 5-FU, both of which are widely used in clinical practice.

When administered individually, cisplatin, 5-FU and SHIN1 exhibited only modest inhibitory effects, with cell viability remaining comparable to that of the control. In contrast, DS18561882 demonstrated a substantial reduction in viability, inhibiting ~50% of cell growth. Notably, combination treatment with either SHIN1 or DS18561882 and cisplatin led to a significantly enhanced inhibitory effect compared to each agent alone (Fig. 6B, Supplementary Fig. 3A). Further synergistic analysis using the Highest Single Agent (HSA) and Bliss Independence model confirmed synergistic interactions between cisplatin and both 1C metabolism inhibitors (Fig. 6C, D) [53, 54]. In contrast, no synergistic effect was observed when either inhibitor was combined with 5-FU (Supplementary Fig. 3B, C). In conclusion, our data implies that targeting the *CD44*-1C metabolism axis synergizes with cisplatin in *CD44*-high intestinal-type GC. The validation in organoids suggests that these findings could lead to the development of more effective and clinically applicable therapies, offering a new therapeutic strategy beyond conventional chemotherapeutics.

## Discussion

In our study using the PDO model, we identified significant changes that highlighted ITH. This finding was further validated with human data, confirming that PDOs can be effectively used as a preclinical platform with potential clinical and therapeutic applications.

We demonstrated ITH across four distinct regions within two different GC subtypes. Notably, one clonal sample from the intestinal-type, which was expected to have relatively low stemness, showed high expression of cancer stem cell markers, underscoring the presence of ITH. Specifically, single-nucleus transcriptome profiling revealed that one clonal sample from the intestinal-type PDO exhibited stemness levels similar to those of the diffuse-type. These results were consistent with data from single-cell transcriptome analysis of human GC tissue. Although our analysis was conducted on organoids rather than human tissue, the absence of information on factors like the tumor microenvironment remains a significant limitation. This has raised ongoing questions about the extent to which PDOs can accurately represent the outcomes observed in human tissues. However, recent studies on the representativeness of PDOs, combined with the findings of this study, strongly suggest that PDOs can effectively mirror the data obtained from human tissues [31, 55–57].

Another significant discovery was the genomic variation found within clonal samples of the same histological type. For example, among the intestinal-type GC141 clonal samples, *CD44* was highly expressed in only one sample, highlighting the importance of genetic profiling. This finding aligns with recent research that underscores the need for detailed genotypic classification beyond traditional histological categories [58].

Research has established that higher stemness in cancer is linked to greater malignancy and chemotherapy resistance, with *CD44* serving as a key marker for cancer stem cells [59–63]. Recent studies have also revealed a strong connection between stemness and 1C metabolism, which is involved in nucleotide metabolism, NADPH balance, lipid metabolism, ROS regulation, and epigenetic methylation. These processes are critical in cancer, particularly in promoting tumor growth [28, 29, 64–67]. In our data, the intestinal-type sample with high stemness also exhibited increased 1C metabolism, further supporting the link between stemness and 1C metabolism and revealing a positive correlation [68–72]. Additionally, survival data from the TCGA database showed that patients with high expression of both *CD44* and 1C metabolism-related genes tended to have poorer outcomes.

Notably, the observed inhibitory effects of 1C metabolism inhibitors in *CD44*-high PDOs highlight their therapeutic relevance, particularly given their superior efficacy compared to conventional chemotherapeutics. This finding underscores the potential of targeting the *CD44*-1C metabolism axis as a novel strategy in intestinal-type GC. Intriguingly, synergistic effects were only detected with specific chemotherapeutics, suggesting that synergistic interactions are mechanism-dependent and may not universally apply across all drug classes. The absence of synergistic effect with 5-FU further emphasizes the need to prioritize drug combinations aligned with the molecular context of the tumor.

**Fig. 7:**
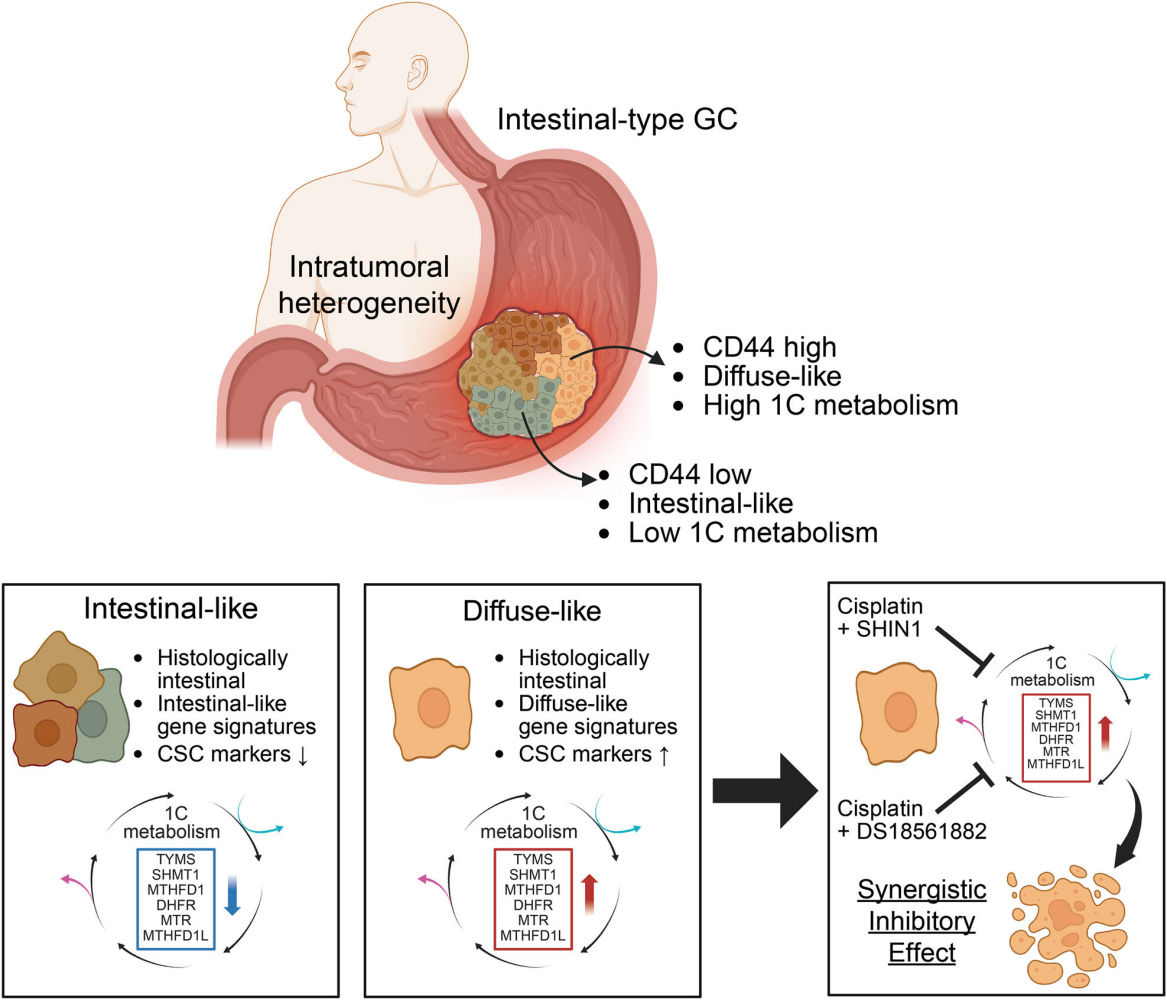
Schematic diagram illustrating molecular mechanisms and targeted pathways to enhance therapeutic efficacy in diffuse-like intestinal-type gastric cancer. Intestinal-type gastric cancer displays intratumoral heterogeneity (ITH), with diffuse-like subpopulations exhibiting high *CD44* expression and elevated 1C metabolism. Combining conventional chemotherapy with 1C metabolism targeting results in a synergistic inhibitory effect on tumor growth, offering a promising strategy for *CD44*-driven GC subtypes (SHIN1:SHMT1/2 inhibitor; DS18561882: MTHFD1/2 inhibitor).

In summary, our research offers important perspectives on the intricate connection between ITH and the effectiveness of PDOs in GC studies. While histological information is crucial for treatment decisions in solid cancers, including GC, our findings highlight the importance of assessing 1C metabolism-related genes in intestinal-type GC patients with high *CD44* expression (Fig. 7) [73, 74]. We propose that targeting these pathways with specific inhibitors could lead to the development of novel therapeutic strategies.

## Materials and methods

### Sample collection

The GC specimen was obtained through surgical resection. The resected stomach was opened laterally to reveal the cancerous lesion, and the multifocal areas of the tumor were designated as C1–C4. The minimum separation between these multifocal regions was 10 mm.

### Generation of patient-derived organoids

Cancer tissues are extracted from the lesion of a stomach cancer patient. Every tissue is washed three times with PBS and minced into ~1 mm pieces. These pieces are then transferred to a 15 mL conical tube and incubated in 8 mL basal medium containing 0.5 mg/mL collagenase type I (Gibco, #17100017) at 37 °C for 90 min. The digested pieces are passed through a 70 μm mesh strainer, and the cell suspension is washed twice with PBS. During the final wash, the pellet is cooled on ice for a short period, then an appropriate volume of Matrigel (Gibco, #A1413202 and Life sciences, #356237) is added. The carefully resuspended pellet is plated in 25 μL each in a 48-well plate and incubated at 37 °C for 1–2 weeks.

Organoids are cultured in 25 μL Matrigel droplets in 48-well plates and subculture every 1–2 weeks. Depending on the experimental purpose, the grown organoids are broken down into small pieces or single cells for use.

### Immunohistochemistry of organoid and primary tumor tissue

Immunohistochemistry (IHC) was conducted using a Ventana XT automated staining instrument (Ventana Medical Systems, Tucson, AZ, USA). The following antibodies were used in accordance with the manufacturer’s instructions: HER-2 (clone 4B5; Ventana, Roche diagnostics, Indianapolis, IN, USA), MutL homolog 1 (MLH1; clone M1; Ventana, Roche diagnostics, Indianapolis, IN, USA), MutS protein homolog 2 (MSH2; clone G219-1129; Ventana, Roche diagnostics, Indianapolis, IN, USA), MutS homolog 6 (MSH6; clone SP93; Ventana, Roche diagnostics, Indianapolis, IN, USA), post-meiotic segregation increased 2 (PMS2; clone A16-4; Ventana, Roche diagnostics, Indianapolis, IN, USA), and *CD44* (1:600; clone MRQ-13; Cell Marque, Rocklin, CA, USA). Tumors classified as microsatellite instability-high or mismatch repair deficient were identified by the absence of one or more proteins (*MLH1*, *MSH2*, *MSH6*, and *PMS2*) through IHC staining.

### Immunofluorescence of organoid

Organoid cells grown in confocal dishes were carefully washed with PBS and fixation was performed with standard methods. Following fixation, the cells were washed three times with PBS for 10 min each. Permeabilization was conducted for 10 min at room temperature using a 0.2% Triton X-100 solution. After a PBS wash, blocking was performed for 1 h at room temperature in 1% BSA. The *CD44* primary antibody (Cell Signaling #3570) was diluted to the appropriate concentration using the same solution as for blocking. The reaction was incubated overnight at 4 °C. A secondary antibody conjugated with Alexa488 or Cy3 was used and reacted for 90 min at room temperature. After staining with DAPI, slides were prepared and observed using an LSM980 confocal microscope.

### Cell viability assay (Organoid)

To assess the viability of organoids, organoid cells were seeded in 100 μL of organoid complete medium containing 5% Matrigel in a 96-well plate. After optimization, 5 × 10^3^ cells per well were selected. The next day, 100 μL of organoid complete medium containing the drugs was added to each well. After 7 days of culture, 10% (20 μL) of WST-8 solution (BIOMAX, #QM2500) was added to each well and incubated for 1–3 h at 37 °C. Luminescence was then measured using a VersaMax ELISA Microplate Reader.

### Single-nucleus RNA sequencing analysis

Processing of the samples was performed by Geninus Inc. (Seoul, Korea; www.kr-geninus.com). After homogenizing the frozen tissue and counting the nuclei, they were isolated using flow cytometry. The 10X Genomics Chromium Instrument, along with the cDNA synthesis kit (10x Genomics: Chromium Next GEM Single Cell 3′ Library and Gel Bead Kit v3.1), was used to create a barcoded cDNA library for single-nuclei RNA sequencing from the sorted nuclei. The quality of the cDNA library was assessed with an Agilent Bioanalyzer. The library was then sequenced using two paired-end 200 bp flow cells on an Illumina NovaSeq 5000/6000 S1 Rgt Kit v1.5 (200 cycles).

### Acquisition of single-cell RNA sequencing data

In addition to the snRNA-seq dataset from organoid clonal samples derived from human GC tissues, single-cell RNA sequencing datasets (GSE150290) from the Gene Expression Omnibus (https://www.ncbi.nlm.nih.gov/geo/) were also used to further explore the characteristics of ITH within different GC subtypes. The public data obtained included samples from both intestinal- (*n* = 8) and diffuse-type (*n* = 15) tumors.

### Bioinformatic analysis

The fastq files were processed using 10X Genomics’ CellRanger software (v6.1.2), with GRCh38 as the reference genome. The R package Seurat (v5.1.0) was employed to create the object, identify cell clusters based on gene expression, and determine markers enriched within each cluster. Variable gene identification was performed using the ‘vst’ method, focusing on 2000 features. Cell clustering was conducted with a resolution of 0.5, and the results were visualized using UMAPs. For differential gene analysis, the ‘FindAllMarkers’ function was utilized with the following parameters: only.pos = TRUE, min.pct = 0.25, and logfc.threshold = 0.25. ‘RunALRA’ was applied to impute missing values for comparing ‘*CD44*_high’ and ‘*CD44*_low’ clusters.

Cluster annotation was performed using the ‘SingleR’ package (v2.4.1), which correlates gene expression profiles of pure cell types with single-cell gene expression data. For cell type annotation in both sequencing datasets, we used the ‘HumanPrimaryCellAtlasData’ function as the reference.

Trajectory analysis was conducted using the ‘Monocle3’ and ‘Slingshot’ package (v2.10.0) to generate detailed pseudotime trajectories for specific cluster subsets [46]. ‘as.SingleCellExperiment’ function was used to convert Seurat objects into SingleCellExperiment objects. Then, lineages were identified, and the trajectories were mapped with UMAP. To assess the correlation between lineage-associated genes, scatterplots with correlation scores were used to visualize their progression along pseudotime. Genes with correlation scores above 0.1 were selected.

Cell–cell communication analysis was performed using the package ‘CellChat’ (v2.1.2) [44]. Communication probabilities were determined by combining the gene expression matrix with established interactions between the signaling of ligands, receptors, and cofactors.

GSEA was performed using the ‘escape’ package (v1.12.0) to identify enriched gene sets in subgroup comparisons [75]. For Hallmark and KEGG pathway analysis, gene sets from the Molecular Signatures Database were utilized [76].

### Statistics

The data are presented as the mean ± standard deviation from three independent experiments. Two-tailed Student’s *t*-test and one-way ANOVA were used to compare values between two groups, with statistical significance set at *P* < 0.05. Data analysis and plot generation were conducted using GraphPad Prism 10 software. Drug synergistic calculations were performed using mathematical formulas derived from SynergyFinder’s HSA and Bliss models.

## Supplementary information

Supplemental Material

## Data Availability

The data that support the findings of this study are available from the corresponding author upon reasonable request.
