# Supplementary material for: One-carbon metabolic pathway is a novel molecular signature for CD44-positive intestinal-type gastric cancer
Source: Cell Death Discov. 2025 Aug 23;11:399. doi: 10.1038/s41420-025-02704-5 (PMC12375131; doi:10.1038/s41420-025-02704-5)
Supplement: Supplementary file 1 — Supplemental Material [file 41420_2025_2704_MOESM1_ESM.docx]

**
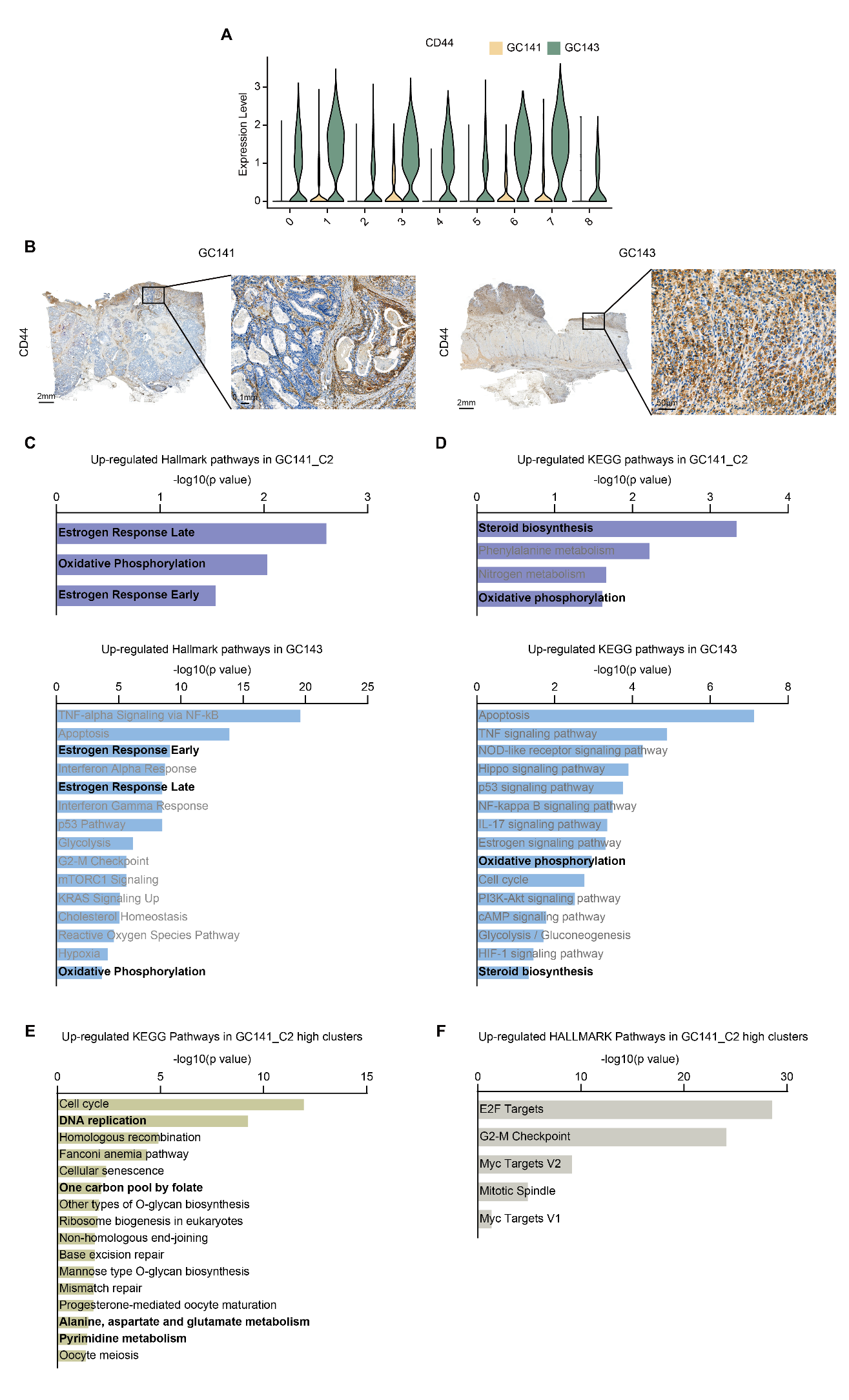
**

**Supplementary Fig. 1 GC141 partially exhibits high *CD44* expression, with GC141_C2 enriching pathways similar to GC143.**

(A) Vlnplot showing *CD44* expression levels of GC141 and GC143, split into clusters. (B) Immunohistochemistry staining of *CD44* performed on tissue samples of GC141 and GC143. (C, D) Comparison of enriched Hallmark and KEGG pathways analyzed using upregulated DEGs from GC141_C2 and GC143, respectively. (E, F) Barplot depicting all KEGG and HALLMARK pathways significantly enriched (p-value below the significance threshold) among upregulated DEGs in the ‘GC141_C2 high’ group.


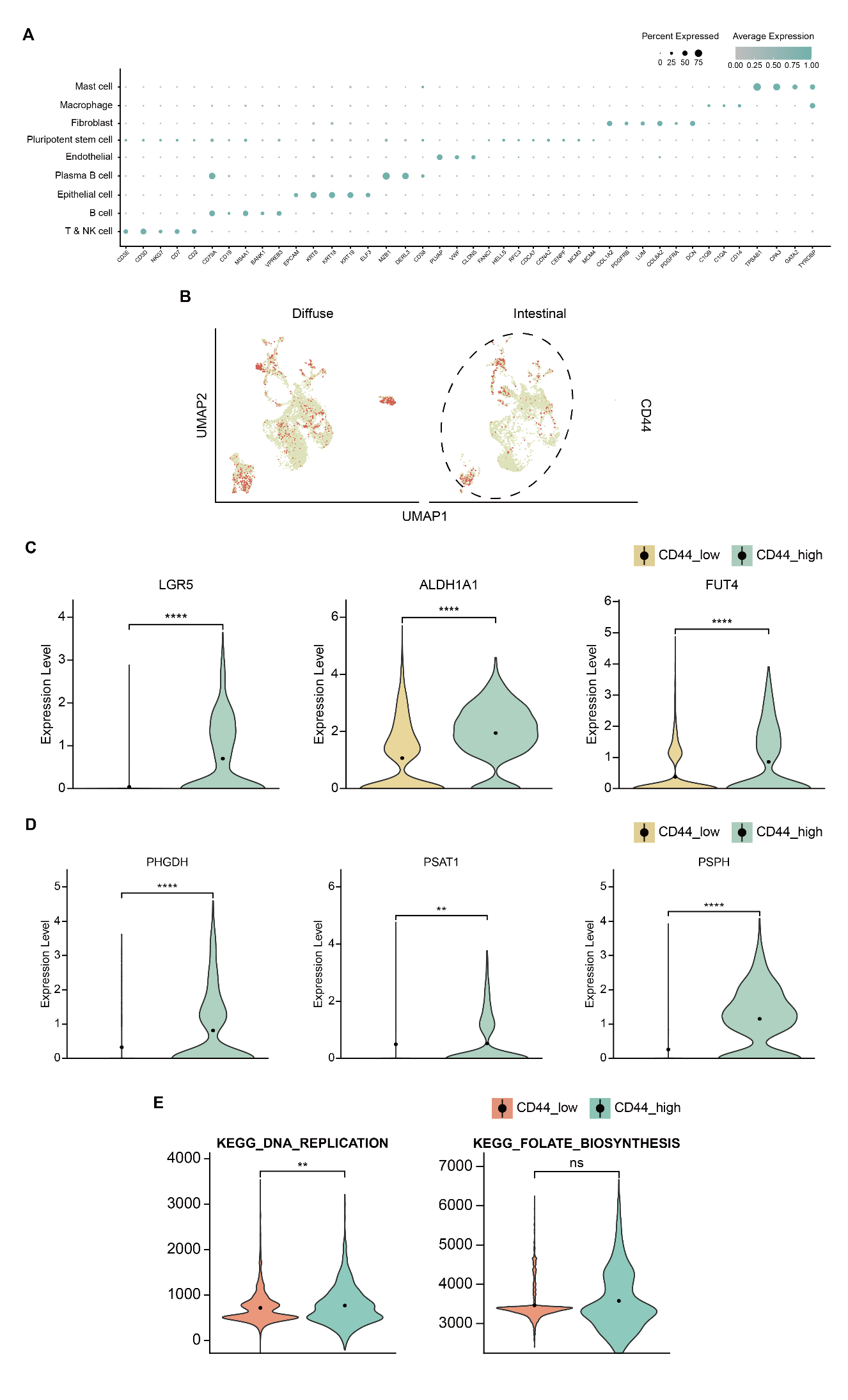


**Supplementary Fig. 2 Clusters with high cancer stem cell gene expression in intestinal-type samples are enriched in 1C metabolism-related pathways.**

(A) Dotplot showing expressions of cell type markers for annotation. (B) Featureplot illustrating *CD44* expression levels of epithelial cell clusters extracted from diffuse- and intestinal-type samples. (C) Vlnplot depicting cancer stem cell-related gene expressions between ‘*CD44*-low’ and ‘*CD44*-high’ clusters of intestinal-type samples. (D) Vlnplot showing serine synthesis pathway-related gene expressions between ‘*CD44*-low’ and ‘*CD44*-high’ clusters of intestinal-type samples. (E) Vlnplot displaying the enrichment of 1C metabolism-related pathways in ‘*CD44*-low’ and ‘*CD44*-high’ clusters of intestinal-type samples. Statistical comparisons were performed using two-tailed Student’s t-test (*P < 0.05, **P < 0.01, ***P < 0.001, ****P < 0.0001, ns, not significant.)­­­.


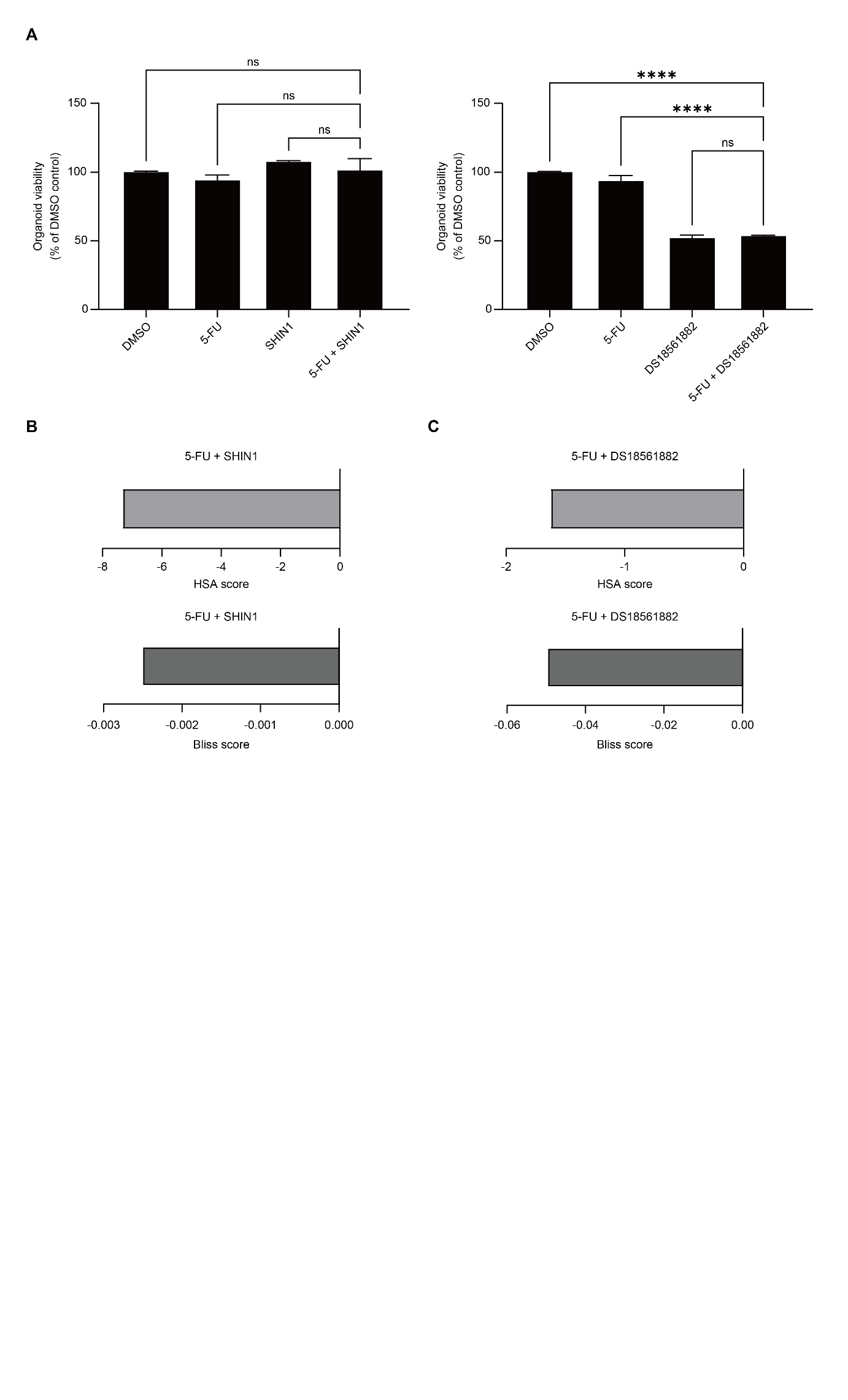


**Supplementary Fig. 3 Patient-Derived Organoid Validation of 1C Metabolism Inhibitors and Their Synergism with Chemotherapeutics**

(A) Organoid viability of GC141_C2 organoid in response to 1C metabolism inhibitors and chemotherapeutic drugs (5-FU 3μM; SHIN1 15μM; DS18561882 40μM). (B, C) The synergistic effects of multiple drugs were validated using the HSA and Bliss models (negative HSA and Bliss scores indicate no synergism). Statistical comparisons were performed using ANOVA (*P < 0.05, **P < 0.01, ***P < 0.001, ****P < 0.0001, ns, not significant).
